# Early life microbiota transplantation from highly feed-efficient broiler improved weight gain by reshaping the gut microbiota in laying chicken

**DOI:** 10.3389/fmicb.2022.1022783

**Published:** 2022-11-18

**Authors:** Abdelmotaleb A. Elokil, Wei Chen, Khalid Mahrose, Mahmoud M. Elattrouny, Khaled F. M. Abouelezz, Hafiz Ishfaq Ahmad, Hua-Zhen Liu, Ahmed A. Elolimy, Mahmoud I. Mandouh, Alzahraa M. Abdelatty, Shijun Li

**Affiliations:** ^1^Key Laboratory of Agricultural Animal Genetics, Breeding and Reproduction, Ministry of Education, College of Animal Science and Veterinary Medicine, Huazhong Agricultural University, Wuhan, Hubei, China; ^2^Animal Production Department, Faculty of Agriculture, Moshtohor, Benha University, Mushthar, Egypt; ^3^Institute of Animal Science, Guangdong Academy of Agricultural Sciences, State Key Laboratory of Livestock and Poultry Breeding, Key Laboratory of Animal Nutrition and Feed Science in South China, Ministry of Agriculture, Key Laboratory of Poultry Genetics and Breeding, Ministry of Agriculture, Guangdong Key Laboratory of Animal Breeding and Nutrition, Guangdong Public Laboratory of Animal Breeding and Nutrition, Guangzhou, China; ^4^Animal and Poultry Production Department, Faculty of Technology and Development, Zagazig University, Zagazig, Egypt; ^5^Department of Poultry Production, Faculty of Agriculture, Assiut University, Assiut, Egypt; ^6^Department of Animal Breeding and Genetics, Faculty of Veterinary and Animal Sciences, The Islamia University of Bahawalpur, Bahawalpur, Pakistan; ^7^Department of Animal Production, National Research Centre, Giza, Egypt; ^8^Department of Nutrition and Clinical Nutrition, Faculty of Veterinary Medicine, Cairo University, Giza, Egypt

**Keywords:** microbial transplantation, early life programming, 16S rRNA gene, body weight gain, laying chicken

## Abstract

Starting phase of laying chicken life is the building stone for rearing and production stages. Since, fecal microbial transplantation (FMT) regulates the gut microbial diversity and affects the productive performance of the bird. The aim of this study is to evaluate the effect of FMT from feed-efficient broiler chicken could program the diversity of gut microbiota and growth of recipient native slow growing egg-laying chicks. For this, a total of 150 (one-day-old) Jing Hong chicks were randomly assigned into two groups, each group consisted of 5 replicates (*n* = 15 bird/ replicate). The control group (CON) and FMT recipient birds (FMT) fed on basal diet, the FMT group received an oral daily dose of FMT prepared from Cobb-500 chickens. The FMT performed from the 1d to 28d of age, through the experimental period, feed intake and body weight were recorded weekly. At the end of a 28-day trial, carcass traits were assessed and cecal samples were collected for microbiome assessment *via* 16S rRNA-based metagenomic analysis to characterize the diversity and functions of microbial communities. The data were statistically analyzed using R software. Body weight and body weight gain increased, and FCR decreased (*p* = 0.01) in FMT group. The relative abundance of *Firmicutes* and the *Firmicutes/Bacteroidetes* (F/B) ratio were increased due to FMT administration (*p* = 0.01). A higher relative abundance of *Lactobacillus*, *Lactococcus*, and *Bifidobacterium* were presented in the FMT group. Meanwhile, *Enterococcus*, *Helicobacter*, and *Bacteroides* were more abundant in the CON group (*p* < 0.01). Kyoto encyclopedia of genes and genomes (KEGG) pathways for microbial functions regarding amino acid metabolism, secondary metabolites biosynthesis, carbohydrate metabolism, energy metabolism, and enzyme families, cofactors, and vitamins were significantly annotated in the FMT group. Overall, FMT administration from the donor of highly feed-efficient broilers improved weight gain by reshaping a distinct gut microbiome, which may be related to the metabolism and health in the recipients laying chicks, providing new insight on the application of the FMT technique for early life programming of laying chickens.

## Introduction

The early maturation of laying chicks and early body weight gain was found to be a key step to achieve the flock uniformity and egg production efficiency (Underwood et al., 2021). One key factor regulating the birds body weight is the microbiota that colonize their gut, the close symbiotic relationship between a host and its gut microbiota affects energy harvest from diet, regulates the host well-being, and promotes the innate and the acquired immunity (Deweerdt, 2014). Additionally, gut microbiota secures the permeability of the intestinal mucosa and regulates the fermentation and absorption of dietary nutrients, which may explain the microbiota importance in the regulation of energy and growth activities (Sonnenburg and Bäckhed, 2016). In our previous studies, we investigated some factors that affect gut microbiota development in chicken, including performance type (Elokil et al., 2020a), medicine (Elokil et al., 2020b), fertility (Elokil et al., 2020a), and diet (Abouelezz et al., 2019). The FMT has been reported as the most effective way for whole microbial community changes, whereas probiotic and antibiotic tools usually alter up to three orders of the 100 trillion native microorganisms of the gut (Borody and Khoruts, 2012; Cammarota et al., 2014). The FMT, also known as stool/fecal transplantation or fecal bacterio-therapy, is the infusion of a liquid filtrate of stools from a donor individual into the gut of a recipient individual to modulate the microbial community for therapeutic or medical purposes (Zhang et al., 2012) and to establish a durable alteration of the recipient’s gut microbiota (Grehan et al., 2010).

The microbial Firmicutes/Bacteroidetes (F/B) ratio is important with positive correlation of somatic growth, as these taxa include bacteria that ferment indigestible carbohydrates providing an extra source of energy for the host, which is eventually stored in skeletal muscle and adipose tissue and used for somatic growth (Cornejo-Pareja et al., 2019). Body weight selection was shown to result in quantitative genetic-correlated responses in gut microbiota and in significant differences in heritability of the F/B ratio, and the genetic correlations between high and low body weight chickens were estimated (Meng et al., 2014). Moreover, the higher F/B ratio of gut microbiota has been associated with overweight in humans (Karvonen et al., 2019), mice (Vadder de and Mithieux, 2018), and rabbits (Zeng et al., 2015). The gut microbiota of broiler chicks, which have a high F/B ratio, were found to have higher ability to ferment diet ingredients to produce volatile fatty acids than that of laying chicks (Walugembe et al., 2015). This suggests that a high F/B ratio in the gut microbial diversity and/or richness is associated with body weight gain. On the other side, the intentional change of gut microbiota through fecal microbiota transplantation (FMT), probiotics, and antibiotics has been used for several purposes including improvement of bird’s growth performance.

Jing Hong chicken was selected for this study as it is a prominent native breed in China, which is widely distributed in Hubei Province as one of the important egg varieties with high economic value of egg production. It has a high resistance for endemic diseases, rough feeding conditions, and extensive breeding environment. Therefore, the aim of the present study was to use FMT as a tool to induce early life programming of laying chicks *via* modulation of their gut microbiota, leading to early body weight maturation. Thereby, providing experimental evidence to understand how the intentional programming of gut microbiome affects the growth performance of laying chicks.

## Materials and methods

### Experimental design, diet, and bird management

In a 28-day trial. A total of 150-day-old unsexed of newly hatched chicks from Jing Hong laying strain were randomly allocated into two treatment groups (*n* = 75/group) as follows: (1) birds fed on the basal diet (CON), and (2) birds fed on basal diet and received fecal microbiota transplantation (FMT). Each dietary treatment consisted of 5 replicates (15 birds/ replicate). The chicks were housed in growth metal cages in an enclose poultry farm of Huazhong Agricultural University throughout the study period, each cage was equipped with one manual feeder and drinker to ensure the animals’ nutritional requirements. A tray with a parchment paper was placed under each cage for fecal sample collection. The diet was formulated following the guidelines of Chinese Feeding Standards using the Feed database in China (Beijing, 2004), diet ingredients and nutrient composition is provided in Table 1. All birds were fed on the same iso-nitrogenous and iso-caloric diet and water was provided *ad-libitum*.

**Table 1:** Composition and nutrient levels of experimental diet (% as fed-basis).

Ingredients*	% (As-Fed)
Corn	65.0
Soybean meal	11.0
Corn gluten meal	2.70
Corncob powder	13.68
Lard	3.00
*L*-lysine HCl	0.42
*DL*-Methionine	0.22
*L*-Threonine	0.13
Dicalcium phosphate	1.55
Limestone	1.05
Salt	0.25
Premix^1^	1.00
Chemical analysis (calculated)	
Crude protein	13.00
Lysine	0.83
Methionine + Cysteine	0.66
Calcium	0.79
Non-phytate phosphorus	0.34

1 Provided the following per kilogram of diet: Vitamin A, 6000 IU; Vitamin D_3_, 500 IU; Vitamin E, 20 IU; Vitamin K_3_, 0.50 mg; Vitamin B_1_, 2.1 mg; Vitamin B_2_, 3.0 mg; Vitamin B_6_, 3.5 mg; Vitamin B_12_, 0.01 mg; pantothenic acid, 10 mg; niacin, 15 mg; biotin, 0.15 mg; folic acid, 0.45 mg; choline chloride, 500 mg; Fe, 80 mg; Cu, 7 mg; Mn, 60 mg; Zn, 65 mg; I, 0.35 mg; Se, 0.23 mg.

* Metabolizable Energy (12.06 MJ/kg diet).

### Preparation and inoculation of the FMT material

Seventy-five newly hatched Cobb-500 broiler chicks were used daily as a stool donor. The FMT was prepared following the methods (Matsuoka et al., 2014). Briefly, 10 g of fresh fecal samples were collected daily in the morning into the sterile tube (50 ml). The white part of the excreta was removed immediately, because it mainly comprises uric acid, and the fecal part was mixed with 0.75% saline in 1:6 ratios (6 ml of 0.75% saline for each gram of feces) and span at low speed (800 *× g*) for 3 min at 4°C to separate undigested feed and particulate material from the microbial fraction. Keeping the mixture on ice until precipitates were fully settled down, the supernatant was collected and filtered with the sterile gauze to get fecal suspension.

### Performing FMT in laying chickens

A total of 150 females of the recipients Jing Hong chicks similar age with the donor Cobb-500 (one-day-old) were randomly assigned into two groups (FMT and CON), each group consisted from 75 chicks into 5 replicates (*n* = 15 bird/ replicate). Each laying chick in FMT group was provided with an oral fresh dosage of 1 ml of FMT solution daily from day 1 to day 28 of age, at early morning and before having access to feed and water. The transplant was orally administered using 1-ml oral dosage *via* oral-syringes with soft flexible tips that were positioned at the back of the chicks’ tongues. The chicks were then supervised to ensure that they had swallowed the material and feed was with-held for 60 min after administration.

### Growth performance and carcass traits

Live body weight was recorded weekly and used to determine the weekly body weight gain and feed intake was recorded weekly, FCR was then calculated. At 28 days of age, 11 healthy chicks of group average BW were selected and euthanized. The chicks’ breasts (pectoralis major) and thigh (drumstick) muscles were skinned, de-boned and weighted; their livers weight, and the lengths (cm) of their duodenum, jejunum, ileum, and ceca were measured similar to Abdelatty et al. (2021).

### Microbial DNA extraction

To obtain samples for microbial genes analysis, fresh samples from the cecum (*n* = 11/ group) were aseptically collected 5–10 min after slaughter under anaerobic conditions and placed into sterile 2-ml cryo-tubes (Sarstedt, Nümbrecht, Germany), then, samples were immediately stored at −80°C until total microbial DNA extraction. Then, all the sample tubes were instantaneously snap-frozen in liquid nitrogen, and subsequently stored at −80°C for extraction of total microbial DNA later.

The microbial DNA was extracted from 300-μl cecal samples using a DNA stool mini kit (QIAmp DNA Stool mini Kit; QIAGEN, Hilden, Germany) following the manufacturer’s instructions. A Qubit 2.0 Fluorometer (Thermo Fisher Scientific Co., Ltd., Shanghai, China) with a Qubit dsDNA HS assay kit (Life Technologies) was used to quantify DNA concentration.

### PCR amplification and 16S rRNA sequencing

An aliquot of each of the extracted DNA samples was used as a template for PCR amplification. Additionally, 16S ribosomal RNA, library preparation, and DNA sequencing were performed by a commercial provider (Personalbio Co., Ltd., Shanghai, China). To generate an approximate amplicon size of 570 bp, primers (forward 5′ CCTAYGGGRBGCASCAG GNG 3′, reverse 5′ GGACTACNNGGGTATCTAAT 3′) targeting V3-V4 region amplicons were used for amplification and PCR products were purified (Busato et al., 2022; Tawfik et al., 2022). The PCR conditions were as follows: initial denaturation, annealing, and extension were carried out and repeated at 94°C for 4 min, 94°C for 30 s, 50°C for 45 s, and 72°C for 30 s for 25 cycles.

### Sequence quality control and calculation of operational taxonomic units (OTUs)

For each library, amplified libraries of V3-V4 region amplicons were pooled and sequenced using the Illumina Miseq 2000 platform sequencer, including 250-bp paired-end reads generated with a 7-cycle index read (sequences with an overlap longer than 10 bp without any mismatch were assembled). The resultant overlapping paired-end reads were stitched and quality filtered using Microsynth after removing barcode and primer sequences. To perform sequence quality control, the pre-filtered and stitched reads were processed using the QIIME software (Quantitative Insights into Microbial Ecology, v1.8.0).^1^ Briefly, the raw tags were merged based on the overlap of two reads. Then, clean tags were created by pretreating the raw tags and removing the chimeric sequences to generate effective tags. Finally, quantitative analysis of the sequences was performed using the QIIME software, and the chimeric sequences were eliminated using the USEARCH software (v5.2.236).^2^

### Bioinformatics analysis

Quality sequences were counted for each sample after the removal of the chimeric sequences (shorter than 160 bp), and statistical estimations were created for the distribution of sequence length using the R software to characterize the length distribution of the sequences contained in each sample. Then, sequences were clustered into OTUs with 97% similarity using open-reference OTU picking and UCLUST (Edgar, 2010). In addition, Specaccum analysis was applied to check if all sample sizes and the OTU abundance matrix were sufficient to estimate community richness (Supplementary Figure S1). Finally, the taxonomy of OTUs was determined using the Greengenes default database in QIIME (DeSantis et al., 2006). The most abundant OTUs between groups (CON and FMT) were then classified against the NCBI nucleotide database using BLASTN for taxonomic classification, targeting the 16S rRNA marker. For the analysis of bacteria and archaea, the databases for the 16S rRNA gene, Greengenes (Release 13.8)^3^ and RDP (Ribosomal Database Project, Release 11.1),^4^ were used by default to identify the OTU diversity among the samples and between the groups. A Venn diagram was constructed to calculate the total number of OTUs per sample (i.e., group) using the R software.

### Annotation of microbial composition

A rarefaction depth of 12,000 sequences was used to perform α- and β-diversity analyses among samples; the α-diversity was determined using the unweighted and weighted UniFrac distance (Lozupone et al., 2007). The analysis of α-diversity (Shannon, Simpson, Chao1, and ACE indices) was applied to find the drivers of variation in the microbial community structure in the samples (Shannon, 1948; Simpson, 1949; Chao and Shen, 2004). In addition, rarefaction curves of β-diversity (PCA, PCoA, NMDS, and UPGMA cluster analyses) in the samples were calculated using a maximum rarefaction depth of 12,000 sequences and the observed OTU index (Ramett, 2007). The orders of phylogenetic tree construction (MEGAN and KRONA) were used for microbial interactive visualization and to visually display the results of species annotation between groups based on OTU tags. Then, a heat map was constructed according to the distribution of the top 50 abundances and the degree of similarity between the samples. The order analyses of PLS-DA, LDA, PERMANOVA, and ANOSIM were performed to determine the variation in community structure between groups for screening key species.

### Annotation of microbial function prediction

To predict the metabolic functioning of bacteria based on total genome sequences from the 16S rRNA gene, Kyoto Encyclopedia of Genes and Genomes (KEGG) function annotation of the sequence was carried out based on Tax4Fun, then visualized using the statistical analysis of metagenomic profiles (STAMP) software package (Langille et al., 2013). KEGG orthologies (KOs) were categorized into KEGG level 2 pathways with the removal of the non-microbial pathways. The classified genes were divided into six categories, namely, Metabolism, Genetic Information Processing, Environmental Information Processing, Cellular Processes, Organismal Systems, and Human Diseases, each of which was further divided into multiple levels. The nearest sequenced taxon index (NSTI) was used to provide a measure of the availability of sequenced reference genomes for each OTU in a sample. It calculates the average branch length for each sample between an OTU and the nearest sequenced reference genome based on the Greengenes reference phylogeny, accounting for OUT abundance. Finally, the R software was used for the cluster analysis of the top 50 most abundant functional predictions in each sample, presented by the heat map (Langille et al., 2013).

### Statistical analysis

The analysis of β-diversity included PCA and PCoA, which were calculated using weighted UniFrac and unweighted UniFrac. One-way ANOVA and the LM procedure of the R software version 3.2.2, R Core were applied to estimate the different bacterial taxa in CON and FMT groups (R Development Core Team, 2015). Each chick individual was considered an experimental unit, and FMT was included as a fixed effect in the statistical model. All differences were considered significant at *p* < 0.05 and were considered trends when *p* < 0.10. Pairwise comparison was performed using T tests.

## Results

### Growth performance and carcass traits

The differences in body weight, daily weight gain, internal organs weight, and length of the digestive tract sections between CON and FMT groups of laying chickens are shown in Table 2. FMT increased final body weight (*p* < 0.01) by 2.5% and average daily gain increased (*p* < 0.01) by 2.3%. Feed conversion ratio was lower in FMT group compared with the CON group (*p* = 0.01), breast muscle weight increased by 9.7% and thigh muscle weight increased by 17.63% in FMT chickens compared with the control counterpart (*p* = 0.01). Liver weight increased by 8.5% (*p* = 0.01). On the other side, duodenum, ilium, and cecum length was shorter in MFT group compared with the CON one (*p* < 0.05).

**Table 2:** Effect of oral fecal microbiota transplantation from the donor commercial broiler chickens to the recipient native Jing Hong laying chickens on body weight and some carcass traits^1^.

Item	Control	FMT	SEM	*P*-Value^*^
Initial body weight, *g*	29.87	30.75	0.44	0.19
Final Body weight, *g*	187.90	192.50	3.81	0.01
Average daily gain, *g*	5.64	5.77	0.13	0.01
Feed intake, g/d	22.27	22.29	0.32	0.12
Feed conversion ratio	3.98	3.84	0.15	0.01
Breast muscle weight, *g*	5.98	6.56	0.27	0.01
Thigh muscle weight, *g*	7.60	8.94	0.38	0.01
Liver weight, *g*	4.58	4.97	0.17	0.21
Duodenum length, *cm*	16.40	14.77	0.47	0.12
Jejunum length, *cm*	49.00	50.54	1.69	0.13
Ileum length, *cm*	6.77	6.45	0.24	0.12
Cecum length, *cm*	6.40	5.95	0.24	0.11

1 Values are mean ± SEM. Samples number (*n* = 11/ group).

* *P* ≤ 0.05 considered significant.

### 16S rRNA sequencing metrics and quality

High-throughput sequencing generated from 22 chickens (*n* = 11/ dietary treatment) yielded a total of 775,945 reads (average of 35,270 and range of 32,836–38,767 reads per sample; Supplementary Figure S2). Average read length was 160 bp, and the distributions of sequence lengths shown in OTUs were generated based on the greengene database using quantitative insights into microbial ecology (QIIME) and characterized for different taxonomic levels including domain, phylum, class, order, family, and genus. The taxa considered as common in the samples were used in further analysis. Statistical number of OTUs at each classification level among groups of control and antibiotics-exposed chicks are presented in Supplementary Table S1. A total of 12 phyla, 23 classes, 35 orders, 60 families, 77 genera, and 31 species were identified in these samples Supplementary Table S2.

### Alpha and beta diversities

Total observed OTU count, α indexes (ACE and Chao1), and diversity indicators (Simpson and Shannon) between groups are summarized in Table 3. The total observed OTUs was significantly increased (*p* < 0.01) in the FMT group than in the CON group. In contrast, the means of ACE and Chao1 were significantly decreased (*p* < 0.01) in the FMT group than in the control group. Similarly, the Simpson and Shannon indices were significantly decreased in the FMT group than in the CON group. In addition, β-diversity indicators, principal component analysis (PCA), non-metric multidimensional scaling (NMDS), and principal coordinate analysis (PCoA) were obtained to measure the between-group and inter-group distance of the CON and FMT groups.

**Table 3:** Observed operational taxonomic units (OTUs) and α-diversity measures of bacterial communities between chicks from the control and fecal microbiota transplantation (FMT) groups.

Alpha diversity index	Control	FMT	SEM	*P*-Value
Observed OTUs	4357.68	6129.37	311.26	0.0
*Abundance index*				
ACE	1227.56	794.68	66.82	0.03
Chao1	1041.05	770.13	56.09	0.02
*Diversity index*				
Simpson	0.938	0.884	0.016	0.01
Shannon	6.714	5.51	0.24	0.01

All data are expressed as the mean ± SEM (*n* = 11/group) and presented for a similarity of 0.97 between the reads. Values between groups mean the index differ significantly (*p* ≤ 0.05).

**Figure 1:**
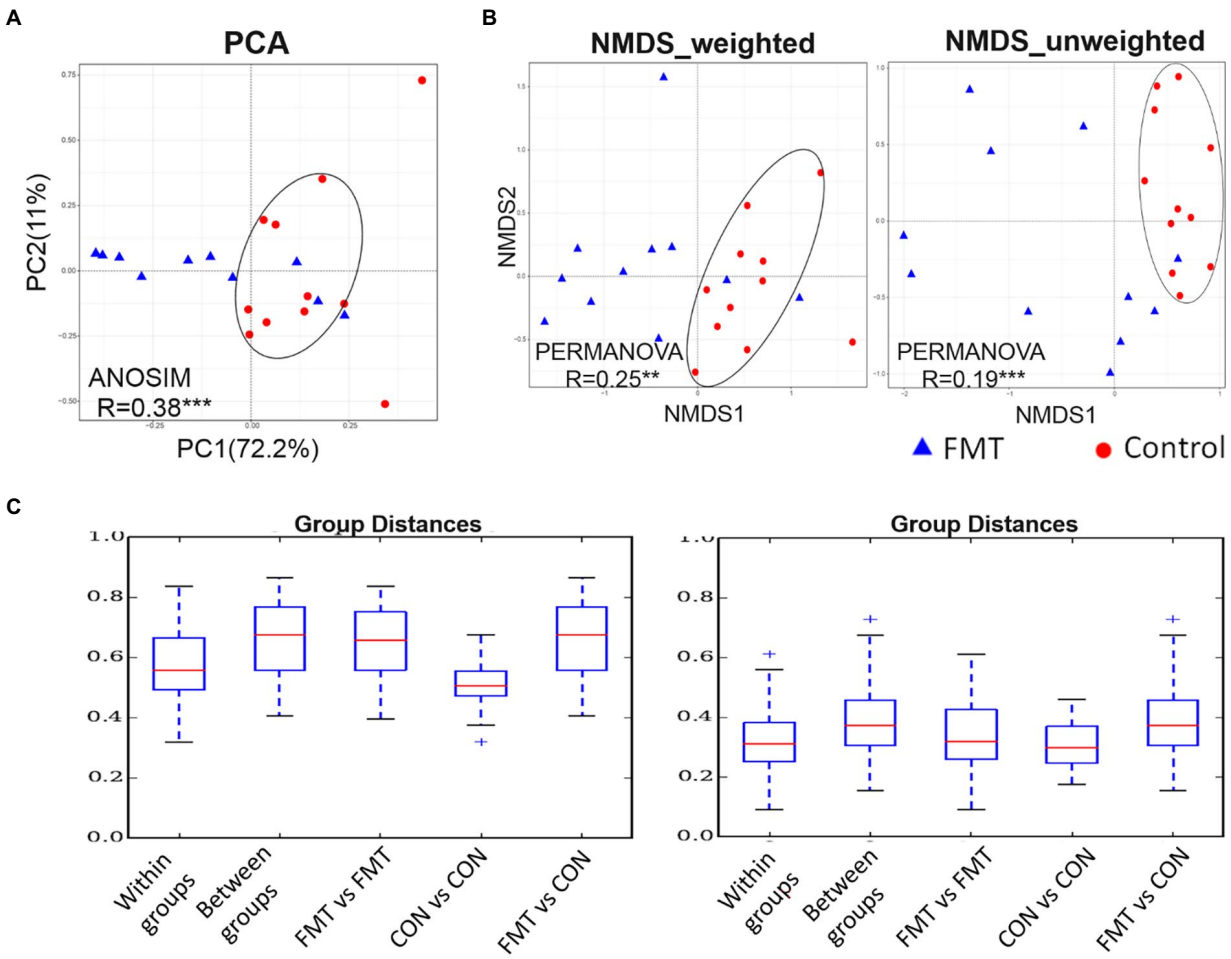
The PCA (principal component analysis) based on unweighted UniFrac distance. **(A)** the PCA, **(B)** non-metric multidimensional scaling (NMDS) of weighted and unweighted of unifrac distance, and **(C)** boxplot for comparative analysis of inter-group/group differences in unifrac distance between groups of control and FMT chicks. Two independent cluster showed significant correlated by ANOSIM (R = 0.38^***^) and PERMANOVA of weighted (R = 0.25^**^) and unweighted (R = 0.19^***^) of unifrac distance between control and FMT of laying-chicks.

The PCA based on unweighted UniFrac distance is presented in Figure 1. The PCA plot revealed that the distance between samples of the CON group were smaller than that of the FMT group, indicating that FMT had a more diverse gut microbiota. The R value of the analysis of similarities (ANOSIM; 0.38) was correlated among samples within the CON and FMT groups (*p* < 0.01). Similarly, the weighted and unweighted values of NMDS indicated that samples of the CON group formed a tighter cluster than those of the FMT group. Regarding the R value obtained from the mutational multivariate analysis of variance (PERMANOVA), there were significant correlations in weighted (R = 0.25; *p* < 0.05) and unweighted (R = 0.19; *p* < 0.01) UniFrac distances among samples and between groups of the CON and FMT groups. In addition, the lowest group distance was significantly estimated (*p* < 0.05) within groups in the comparative analysis boxplot.

### Microbial structure and composition

**Figure 2:**
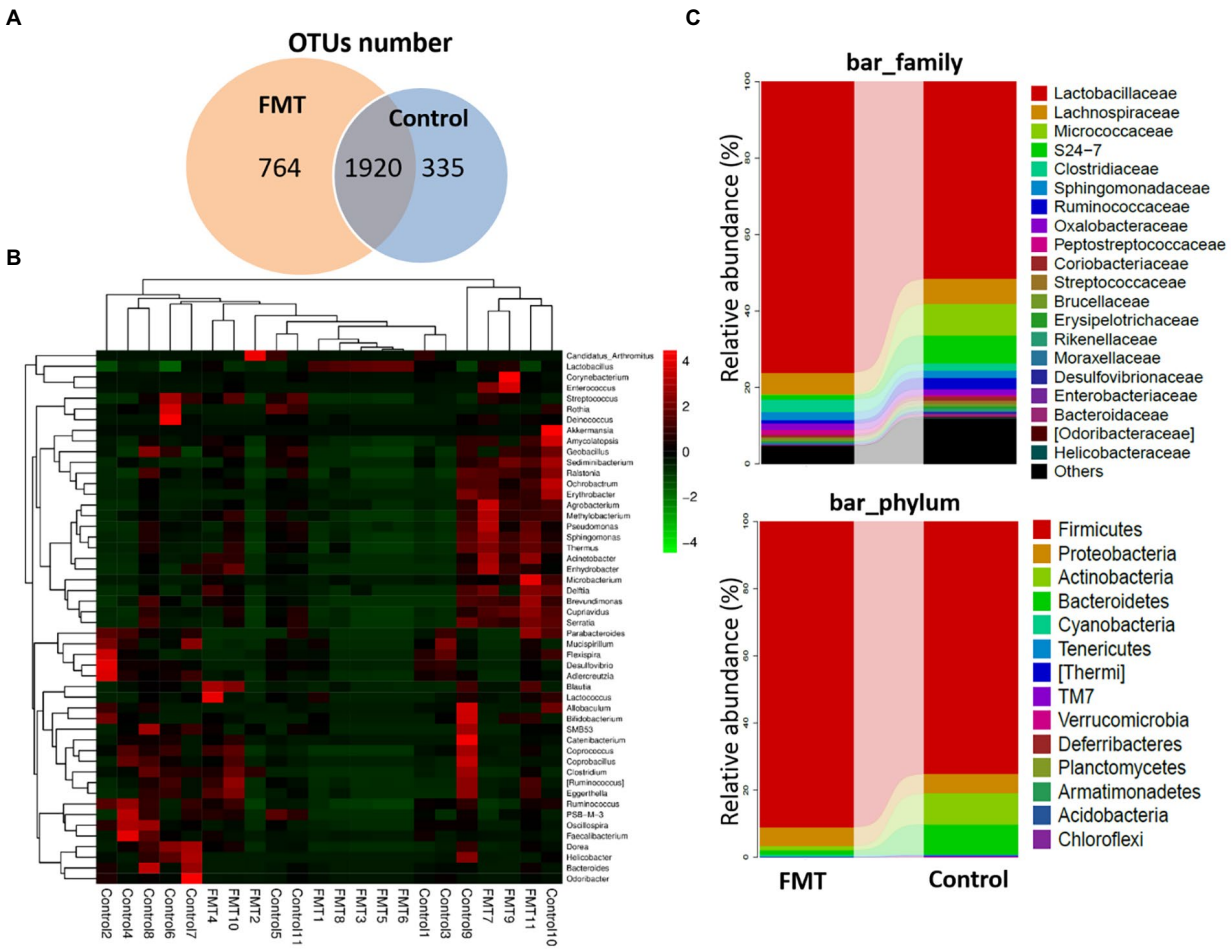
Impact of FMT on the Chickens Gut Microbiota Structure. **(A)** Venn diagram of OUT number between groups (Control and FMT) of common and unique units. **(B)** Heat map showing the genera with significant differences of relative abundances between groups. **(C)** Taxonomic composition analysis between groups at each classification level of phylum and family control and FMT groups.

Figure 2 illustrates the effect of FMT on gut microbiota structure of Jing Hong chickens. The number of OTUs was significantly increased (*p* < 0.01) in the FMT group, with a high abundance of unique OTUs when compared with the CON group. Additionally, a heat map of the top 50 most abundant genera in the microbiome community combined with a cluster analysis revealed similar microbiome composition among samples of the CON and FMT groups. The differences between the CON and FMT groups regarding the relative abundance (percent of total sequences) of each phylum and family of taxa from the microbiome are presented in Figure 2 and Table 4.

**Table 4:** Relative abundance (percent of total sequences) of taxa in the cecal microbiota of chicks from the control and fecal microbiota transplantation (FMT) groups estimated using metagenomic analysis^1^.

Taxa levels	Bacteria Classification	Control	FMT	SEM	*P*-Value
Phylum	*Firmicutes*	75.39	89.05	1.690	0.001^***^
Genus	*Lactobacillus*	9.12	17.87	2.270	0.003^**^
Genus	*Lactococcus*	0.035	1.75	0.090	0.001^***^
Genus	*Enterococcus*	0.86	0.55	0.410	0.023^*^
Genus	*Ruminococcus*	1.34	0.86	0.460	0.041^*^
Genus	*Facklamia*	0.17	0.11	0.060	0.555
Genus	*Arthromitus*	0.16	0.14	0.040	0.721
Genus	*Erysipelothrix*	0.23	0.41	0.180	0.037^*^
Phylum	*Bacteroidetes*	5.80	2.51	0.690	0.003^**^
Genus	*Bacteroides*	1.30	0.54	0.170	0.006^**^
Genus	*Parabacteroides*	0.40	0.26	0.060	0.025^*^
Genus	*Prevotella*	0.609	0.033	0.008	0.001^***^
Genus	*Alistipes*	0.081	0.107	0.019	0.070
Genus	*Barnesiella*	0.111	0.054	0.019	0.042^*^
Phylum	*F/B ratio*	12.99	35.45	1.376	0.001^***^
Phylum	*Actinobacteria*	0.736	0.402	0.146	0.023^*^
Genus	*Bifidobacterium*	0.051	0.064	0.019	0.044^*^
Genus	*Collinsella*	0.001	0.002	0.000	0.557
Genus	*Corynebacteriaceae*	0.004	0.002	0.001	0.444
Genus	*Nocardiaceae*	0.0004	0.0009	0.0003	0.318
Phylum	*Campilobacterota*	0.811	0.164	0.019	0.001^***^
Phylum	*Proteobacteria*	7.980	3.520	1.881	0.009^**^
Genus	*Comamonadaceae*	1.831	0.654	0.044	0.001^***^
Genus	*Helicobacter*	1.589	1.027	0.018	0.001^***^
Phylum	*Moraxellaceae*	0.019	0.018	0.007	0.946
Genus	*Pasteurellaceae*	0.001	0.001	0.004	0.351
Genus	*Pseudomonadaceae*	0.0004	0.0009	0.000	0.267
Phylum	*Fusobacteria*	0.813	0.897	0.010	0.840
Genus	*Fusobacteriaceae*	0.024	0.037	0.002	0.406
Phylum	*Verrucomicrobia*	1.360	0.740	0.192	0.033^*^
Genus	*Akkermansia*	0.469	0.404	0.068	0.512
Phylum	*TM7*	0.042	0.005	0.008	0.005^**^
Phylum	*Cyanobacteria*	0.094	0.118	0.004	0.547
Phylum	*Other*	7.868	2.579	1.403	0.014^*^

1 All data are expressed as the mean ± SEM (*n* = 11/group) of the percentage of domain bacteria at taxonomic levels (phylum and genus). Data were analyzed using one-way ANOVA. *p*-values between control and FMT chicks were obtained using the t-test. Values between groups are significantly different (^*^p ≤ 0.05, ^**^p ≤ 0.01, ^***^p ≤ 0.001).

At the phylum level, the relative abundances of *Firmicutes* increased and that of Bacteroidetes decreased (*p* < 0.01) in the FMT group, and the *Firmicutes*/*Bacteroidetes* ratio (F/B ratio) increased in the FMT group than in the CON group (*p* < 0.01; Table 4). The differences between the CON and FMT groups regarding the relative abundance (percent of total sequences) of each phylum and family of taxa from the microbiome are presented in Figure 2 and Table 4. At the phylum level, the relative abundances of *Firmicutes* was increased (FMT vs. CON, 89.05 vs. 75.39%) and that of *Bacteroidetes* was decreased (FMT vs. CON, 2.51 vs. 5.80%) in the FMT group (*p* < 0.05). Consequently the *Firmicutes*/*Bacteroidetes* ratio (F/B ratio) increased (FMT vs. CON, 35.45 vs. 12.99%) in the FMT group than in the CON group (*p* < 0.01; Table 4). On the other hand, the relative abundance of *Proteobacteria* (FMT vs. CON, 3.52 vs. 7.98%) and *Campilobacterota* (FMT vs. CON, 0.164 vs. 0.811%) were decreased in the FMT group chickens (*p* < 0.01; Table 4). Similarly, there was decreased (*p* < 0.05) in the relative abundance of phyla of *Verrucomicrobia* (FMT vs. CON, 0.740 vs. 1.36%) and *TM7* (FMT vs. CON, 0.005 vs. 0.042%) in the FMT group than in the CON group as presented in Figure 2 and Table 4.

At the genus level, there were significant increased the relative abundances of some symbiotic taxa such as *Lactobacillus* (FMT vs. CON, 17.87 vs. 9.12%), *Lactococcus* (FMT vs. CON, 2.51 vs. 5.80%) and *Bifidobacterium* (FMT vs. CON, 0.064 vs. 0.051%) in the FMT group compared with the CON group (*p* < 0.01; Figure 2; Table 4). On the other hand, there were significant decreased the relative abundances of some pathogenic taxa such as *Enterococcus* (FMT vs. CON, 0.55 vs. 0.86%), *Helicobacter* (FMT vs. CON, 1.027 vs. 1.589%) and *Bacteroides* (FMT vs. CON, 0.054 vs. 1.30%) in the FMT group in compared with the CON group (*p* < 0.01; Figure 2; Table 4).

### Reshaping gut microbial community by FMT in laying chicks

The results of the LEfSe analysis based on the linear discriminant analysis (LDA) of the specific bacterial taxa that are associated with FMT are presented in Supplementary Figure S3. The LEfSe cladogram representative of the structure of the host–microbiota axis shows a significant shift of microbiota between groups, including a total of 64 bacterial taxa that were significantly different between the CON and FMT groups, with only seven taxa (*Lactobacillallus*, *Lactobacillaceae*, *Lactobacillales*, *Bacilli*, *Dietzia*, *Dietziaceae*, and *Aeriscardovia*) in the FMT group. The LDA score plot presented group-enriched taxa that were significant between the two groups (*p* < 0.05).

**Figure 3:**
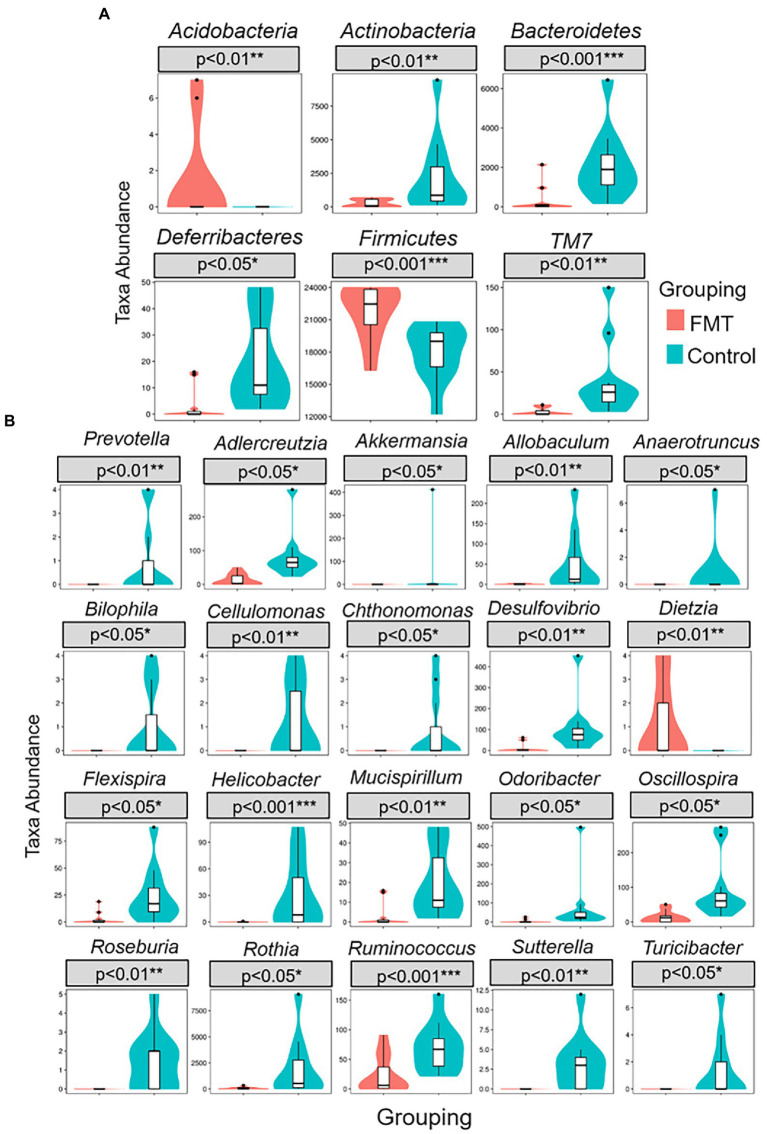
Metastats comparison statistics test for significant differences of taxa abundance between groups of control and FMT chicks. **(A)** six phylum **(B)** twenty genus presented higher F/B ratio in FMT compare with control group.

Figure 3 illustrates the comparison of abundance among groups at the levels of phylum and genus were performed using a Metastats analysis and indicated an increase of the phyla *Firmicutes* and *Acidobacteria* and of the genera *Dietzia* and *Ruminococcus* in the FMT group. The similarity between samples was evaluated by unweighted pair group method with arithmetic mean (UPGMA) analysis, which showed that the samples of the CON and FMT groups were mostly similar and distributed into a different cluster of hierarchical trees (Supplementary Figure S4).

### Microbial function prediction

**Figure 4:**
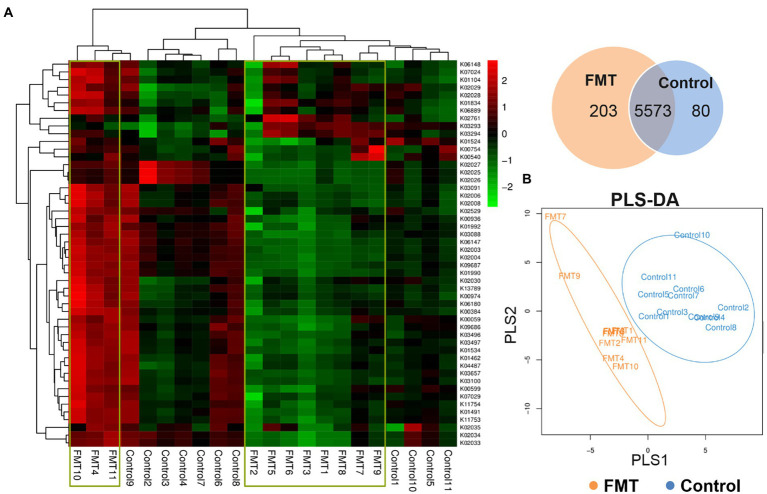
Effect of FMT on Functional Prediction of KEGG Second-level Analysis. **(A)** KEGG orthologous gene cluster abundance heat map combined with cluster analysis based on the similarity of the functional group abundance distribution between samples (red represents a functional group with higher abundance in the corresponding sample, and green represents a functional group with lower abundance); venn diagram of the common and unique functional group, **(B)** PLS-DA discriminant analysis plot showed samples belonging to the same group are closer to each other, and the distance between the points of different groups is farther.

The relative abundance comparison between the CON and FMT groups based on the second-level of KEGG pathways analysis is shown in Table 5 and Figure 4. A total of six categories of cellular processes, environmental information processing, genetic information processing, immune information processing, metabolism processing, and organismal systems processing were revealed between the CON and FMT groups.

**Table 5:** Relative abundance of the functional microbiome predicted by KEGG second-level pathways between chicks from the control and FMT groups estimated using the statistical analysis of metagenomic profiles.

**Categories of KEGG second-level pathways** ^**1**^	**Control**	**FMT**	**SEM**	***P*-Value**
*Cellular Processes*				
Cell growth and death	0.0060	0.0080	0.0000	0.0001^**^
Cell motility	0.0120	0.0180	0.0020	0.0720
Transport and catabolism	0.0010	0.0020	0.0000	0.0001^**^
Unclassified; Cellular Processes and	0.0340	0.0570	0.0040	0.0590^*^
*Environmental Information Processing*				
Membrane transport	0.1280	0.1370	0.0030	0.004^**^
Signaling molecules and interaction	0.0020	0.0010	0.0001	0.006^**^
Signal transduction	0.0130	0.0150	0.0000	0.008^**^
*Genetic Information Processing*				
Folding, sorting and degradation	0.0230	0.0230	0.0002	0.935
DNA replication and repair	0.0890	0.1000	0.0020	0.003^**^
Transcription	0.0090	0.0270	0.0005	0.007^**^
Translation	0.0570	0.0670	0.0010	0.002^**^
Unclassified; Genetic Information	0.0260	0.0250	0.0003	0.035^*^
*Immune Information Processing*				
Immune system diseases	0.0010	0.0010	0.0000	0.616
Infectious diseases	0.0040	0.0030	0.0000	0.041^*^
Metabolic diseases	0.0010	0.0010	0.0000	0.205
Neurodegenerative diseases	0.0010	0.0010	0.0000	0.884
*Metabolism Processing*				
Amino acid metabolism	0.0770	0.0930	0.0030	0.001^**^
Biosynthesis secondary metabolites	0.0020	0.0080	0.0002	0.001^**^
Carbohydrate metabolism	0.1110	0.4510	0.0010	0.004^**^
Energy metabolism	0.0530	0.0910	0.0072	0.032^*^
Enzyme families	0.0170	0.0420	0.0006	0.025^*^
Glycan biosynthesis and metabolism	0.0220	0.0860	0.0003	0.090
Lipid metabolism	0.0290	0.0310	0.0008	0.099
Cofactors and vitamins	0.0340	0.0570	0.0010	0.003^**^
Other amino acids	0.0150	0.0210	0.0003	0.074
Terpenoids and polyketides	0.0080	0.0070	0.0004	0.286
Nucleotide metabolism	0.0470	0.0420	0.0010	0.046^*^
Xenobiotics biodegradation	0.0230	0.0240	0.0010	0.648
Unclassified; Metabolism Processing	0.0230	0.0870	0.0080	0.009^*^
*Organismal Systems*				
Circulatory system	0.0000	0.0000	0.0000	0.717
Digestive system	0.0010	0.0050	0.0000	0.025
Endocrine system	0.0010	0.0060	0.0000	0.001
Excretory system	0.0003	0.0002	0.0000	0.079
Immune system	0.0005	0.0002	0.0000	0.048
Nervous system	0.0010	0.0010	0.0000	0.968
Unclassified; Poorly Characterized	0.0500	0.0480	0.0020	0.003

^1^ All data are expressed as the mean ± SEM of the relative abundance for predicting of functional microbiome; Values between groups are significantly different (*p ≤ 0.05, **p ≤ 0.01, ***p ≤ 0.001). (*n*=11/group).

In the category of cellular processes, the levels of cell growth and death, and transport and catabolism had a higher (*p* < 0.01) relative abundance in the FMT group than in the CON group. The level of membrane transport pathway in the category of environmental information processing was significantly increased (*p* < 0.01) in the FMT group than in the CON group (Table 5). Prediction of functional microbiome in the category of genetic information processing showed that three pathway levels of DNA replication and repair, transcription, and translation were significantly increased (*p* < 0.01) in FMT group. One level of infectious diseases pathway under the category of immune information processing was increased (*p* < 0.05) in the FMT group when compared with the CON group. Regarding the category of metabolism processing, the differences between groups (FMT and CON) in the pathway levels regarding amino acid metabolism (*p* < 0.01), secondary metabolites biosynthesis (*p* < 0.01), carbohydrate metabolism (*p* < 0.01), energy metabolism (*p* < 0.05), enzyme families (*p* < 0.05), and cofactors and vitamins (*p* < 0.05) were higher in the FMT groups than in the CON group. The pathway level of endocrine system was significantly increased (*p* < 0.01) in the FMT group when compared with the CON group.

The top 50 most abundant KEGG orthologous genes were clustered in the heat map combined with the analysis between the CON and FMT groups; samples of the control group formed clusters based on their similar microbial compositions. The Venn diagram of the common and unique functional groups revealed that the FMT group had more functional groups than the CON group. PLS discriminant analysis (PLS-DA) plot showed that samples belonging to the same group were more similar to each other than those from different groups.

## Discussion

There is increasing interest in early life programming of chickens for better health condition and performance. The recent reports showed promising results on the application of FMT in early life of birds (Siegerstetter et al., 2018; Yan et al., 2021) to modulate gut microbiota. Therefore, we investigated the safety of daily dosage of FMT from broiler donor chicks and their ability to alter the composition, structure, and function of gut microbiota and improve metabolic outcomes in the early life of native egg laying chicks with low performance. The gut microbiota of the Cobb-500 broiler (donors) has been previously characterized in several studies, including a distinct microbial community regarding improvement in feed-efficient and weight gain (Siegerstetter et al., 2018; Richards et al., 2019).

In the present study, the dominant phyla were *Firmicutes* and *Proteobacteria* followed by *Bacteroidetes*, *Cyanobacteria*, and *Actinobacteria*; however, the proportion of each phylum fluctuated and was affected by the FMT treatment. We herein observed that *Firmicutes* and the F/B ratio were increased in the FMT group, which was also consistent with an increase in body weight gain of chicks. We therefore speculate that this may be because *Firmicutes* and a high F/B ratio in the cecum microbiota allow a more efficient use of feed energy, since, previous studies have shown that the F/B ratio is associated with mammalian weight gain in different species, such as human (Karvonen et al., 2019), mice (Everard et al., 2014), and rabbit (Zeng et al., 2015). Additionally, the difference between the control group and the FMT group may be attributed to the latter having obtained abundant microbiota from the broiler chicks, which have a higher abundance of microbes in their digestive tract and can thus intake more nutrients.

Moreover, most α-diversity indices (Ace, Chao, Shannon, Simpson) were higher in the control group than in the FMT group, suggesting that the gut microbiota in the control group was more diverse than in the FMT group, which appeared to be more homogeneous based on the overabundance of *Firmicutes* and the F/B ratio. Additionally, in the current study, *Lactobacillus*, *Lactococcus*, and *Bifidobacterium* were more abundant in chicks received FMT. *Lactobacillus* is known to be involved in carbohydrate (Goh and Klaenhammer, 2014) and lipid metabolism (Falcinelli et al., 2015) and in immune system metabolism (Xin et al., 2014). *Lactobacillus* is the most important bacterial genus as it facilitates nutrient absorption, enhances host immunity, and prevents intestinal inflammatory responses (Wang et al., 2018). Collectively, *Lactobacilli* and *Bifidobacteria* are purported beneficial for gut physiology and body weight gain.

Gut microbiota composition is one of the most internal factors affecting physiological responses of the host through host–microbiome interaction, and a normal gut microbiota community allows gut permeability, nutrient digestibility, and anti-inflammatory reactions (Elokil et al., 2020c). Our results suggest that the shift in microbe distribution resulting from FMT successfully improved body weight gain. Therefore, the gut microbiota of the FMT group can digest complex and simple carbohydrates and produce more nutrients, such as volatile fatty acids, microbial proteins, and vitamins, in order for the host to gain weight in comparison with the control group.

Nowadays, CRISPR-Cas technologies, which were developed from the prokaryotic immune system, have recently made it possible for scientists to examine and modify organisms with an unparalleled ease and effectiveness (Vercoe et al., 2013; Ahmed et al., 2018). The delivery of CRISPR-Cas systems to bacterial populations is another option. These systems can be designed to target and destroy particular microbiome components (Ramachandran and Bikard, 2019). Together, these techniques offer fascinating chances to explore the intricate relationships that exist between the microbiome’s constituent parts and human bodies. They also open up fresh possibilities for the creation of medications that specifically target the microbiome (Stout et al., 2018). Most bacteria have CRISPR-Cas systems, which are present in 40% of them. In certain circumstances, it may be possible to use endogenous CRISPR-Cas systems, while in other cases, it may be possible to introduce designed CRISPR-Cas systems into the target bacteria (Hullahalli et al., 2017). The genomes of probiotic or microbiome-associated yeast, bacteria, and bacteriophages can all be altered using these methods. Additionally, based on their sequence, they can be employed to eradicate particular strains without affecting the remainder of the microbiome (Jiang et al., 2013).

Our findings revealed that FMT treatment promoted early body weight gain in Jing Hong chickens, speculating the physical growth will be completed early and will increase the length of laying time in the hens of FMT group than control group (Choi and Cho, 2016). Predictably, cocks in FMT group will have good physical and external morphometric traits such as comb height and color, wattle length, earlobe width, wingspan, girth circumference, drumstick length and testicular weight (Metzler-Zebeli et al., 2016). Since, the body weight- age relationship of laying chicks is directly related to the egg weight and number of egg produced for the laying chickens (Di Masso et al., 1998), and egg weight attains proper flock uniformity which is a key criterion for the success of egg-laying performance (Lacin et al., 2008). Which was achieved in the current study through FMT that regulated gut microbiome uniformity, and increased.

## Conclusion

In conclusion, our findings showed that a distinct microbial community were colonized the gut due to FMT administration from the donor of highly feed-efficient broilers to laying chicks, which improved intestinal functions and antimicrobial pathogenicity compared with the control group. In addition, early life programming of slow growing chicks breed (Jing Hong) by FMT application was successful *via* increasing the cecal abundance of *Firmicutes, Lactobacillus, Lactococcus*, and *Bifidobacterium,* which may be related to increase the carbohydrate metabolism and health in laying chicks. FMT could be a potential strategy to improve animal growth performance.

## Data availability statement

The data presented in the study are deposited in the Genome Sequence Archive (Genomics, Proteomics & Bioinformatics 2021) in National Genomics Data Center (Nucleic Acids Res 2022), China National Center for Bioinformation / Beijing Institute of Genomics, Chinese Academy of Sciences repository, accession number GSA: CRA007995 that are publicly accessible at https://ngdc.cncb.ac.cn/gsa.

## Ethics statement

The animal study was reviewed and approved by The experimental procedures used in this study met the guidelines of the Care and Use of Laboratory Animals of the Standing Committee of Hubei People’s Congress (No. 5) and approved by the Biological Studies Animal Care Committee of Hubei Province P.R. China, and the Ethics Committee of Huazhong Agricultural University, PR China, with approval number HZAUCH-19006. In this study, all efforts were made to minimize the animal suffering and was carried out in compliance with the ARRIVE guidelines.

## Author contributions

AbE, H-ZL, and SL contributed to conception and design of the study and performed the experiment. WC, KA, ME, and KM organized the database. H-ZI, AhE, MM, and AA performed the statistical analysis. SL and WC were partial funding collaborators in this project. All authors equally wrote and revised the manuscript, contributed to the article, and approved the submitted version.

## Funding

This work was supported by Natural Science Foundation of China (No.32072707); Science and Technology Major Project of Hubei Province (2020ABA016); the grants from the National Key R&D Program of China (SQ2021YFD1300002); The Special Fund for Scientific Innovation Strategy-Construction of High Level Academy of Agriculture Science (R2020PY-JX008 and 202106TD); Egyptian Ministry of Scientific Research, The Science, Technology & Innovation Funding Authority (39382). The funders had no role in the design, analysis, interpretation and writing of the manuscript.

## Conflict of interest

The authors declare that the research was conducted in the absence of any commercial or financial relationships that could be construed as a potential conflict of interest.

## Publisher’s note

All claims expressed in this article are solely those of the authors and do not necessarily represent those of their affiliated organizations, or those of the publisher, the editors and the reviewers. Any product that may be evaluated in this article, or claim that may be made by its manufacturer, is not guaranteed or endorsed by the publisher.

## Supplementary material

The Supplementary material for this article can be found online at: https://www.frontiersin.org/articles/10.3389/fmicb.2022.1022783/full#supplementary-material

## Footnotes

^1^ http://qiime.org/

^2^ http://www.drive5.com/usearch/

^3^ http://greengenes.secondgenome.com/

^4^ http://rdp.Cme.msu.edu/
