# Supplementary material for: Early life microbiota transplantation from highly feed-efficient broiler improved weight gain by reshaping the gut microbiota in laying chicken
Source: Front Microbiol. 2022 Nov 18;13:1022783. doi: 10.3389/fmicb.2022.1022783 (PMC9715608; doi:10.3389/fmicb.2022.1022783)
Supplement: Supplementary file 1 [file Data_Sheet_1.pdf]

Frontiers in Microbiology
Supplementary File

Running title: Early life programming of laying chickens using FMT

Early life microbiota transplantation from highly feed-efficient broilers improved weight gain by reshaping the gut microbiota in laying chicken

Abdelmotaleb A. Elokil[1,3] , Wei Chen[2 *], Mahmoud M. Elattrouny[3] , Khalid Mahrose[4] , Khaled F. M. Abouelezz[5] , Hafiz Ishfaq Ahmad[6] , Hua-Zhen Liu[1] , Ahmed A. Elolimy[7] , Mahmoud I. Mandouh[8] , Alzahraa M. Abdelatty[8] and Shijun Li[1 *]

[1] Key Laboratory of Agricultural Animal Genetics, Breeding and Reproduction, Ministry of Education, College of Animal Science and Veterinary Medicine, Huazhong Agricultural University, Wuhan, Hubei, China, [2]Institute of Animal Science, Guangdong Academy of Agricultural Sciences, Guangzhou, China; State Key Laboratory of Livestock and Poultry Breeding, Guangzhou, China; Key Laboratory of Animal Nutrition and Feed Science in South China; Ministry of Agriculture, Guangzhou, China ; Guangdong Public Laboratory of Animal Breeding and Nutrition, Guangzhou, China; Guangdong Key Laboratory of Animal Breeding and Nutrition, Guangzhou, China, [3]Animal Production Department, Faculty of Agriculture, Moshtohor, Benha University, Mushthar, Egypt, [4]Animal and Poultry Production Department, Faculty of Technology and Development, Zagazig University, Zagazig, Egypt, [5]Department of Poultry Production, Faculty of Agriculture, Assiut University, Assiut, Egypt, [6]Department of Animal Breeding and Genetics, Faculty of Veterinary and Animal Sciences, The Islamia University of Bahawalpur, Bahawalpur, Pakistan, [7] Department of Animal Production, National Research Centre, Giza, Egypt, [8]Department of Nutrition and Clinical Nutrition, Faculty of Veterinary Medicine, Cairo University, Giza, Egypt

*For correspondence lishijun@mail.hzau.edu.cn; cwei010230@163.com

Adders: 1 Shizishan Street, Hongshan District, Wuhan 430070, Hubei, P.R. China

Tel. +86-27-873-874-80; Fax: +86-2787280408

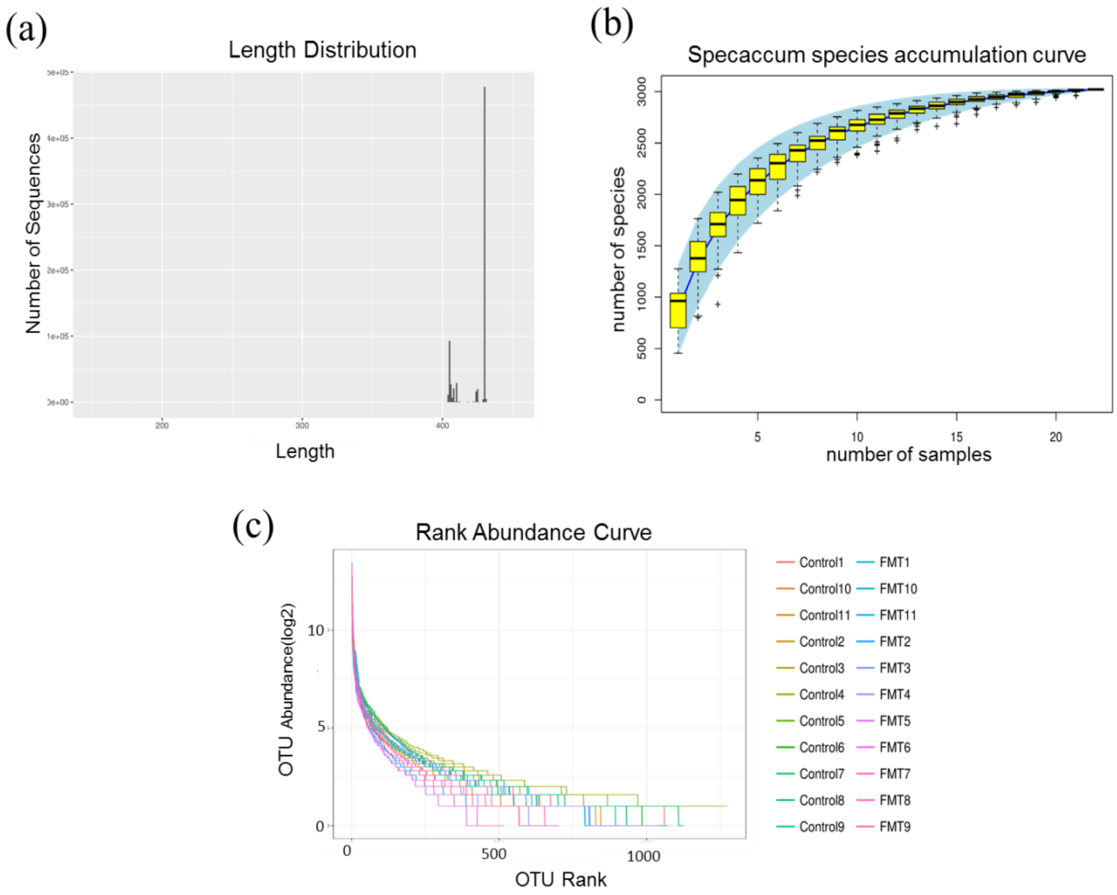

Figure S1. (a) The map of sequence length distribution statistics. The abscissa is the length distribution of the sequence in all samples and the ordinate is the total number of sequences corresponding to each length value. (b) Specaccum species accumulation curve showed that results reflected the rate of increase in new species observed during the continuous sampling of the sample during the overall sampling of the sample. (c) Abundance grade curve visually reflected the number of high abundances and rare OTUs in the community through abundance value was converted into the ordinate by Log2 logarithmic transformation.

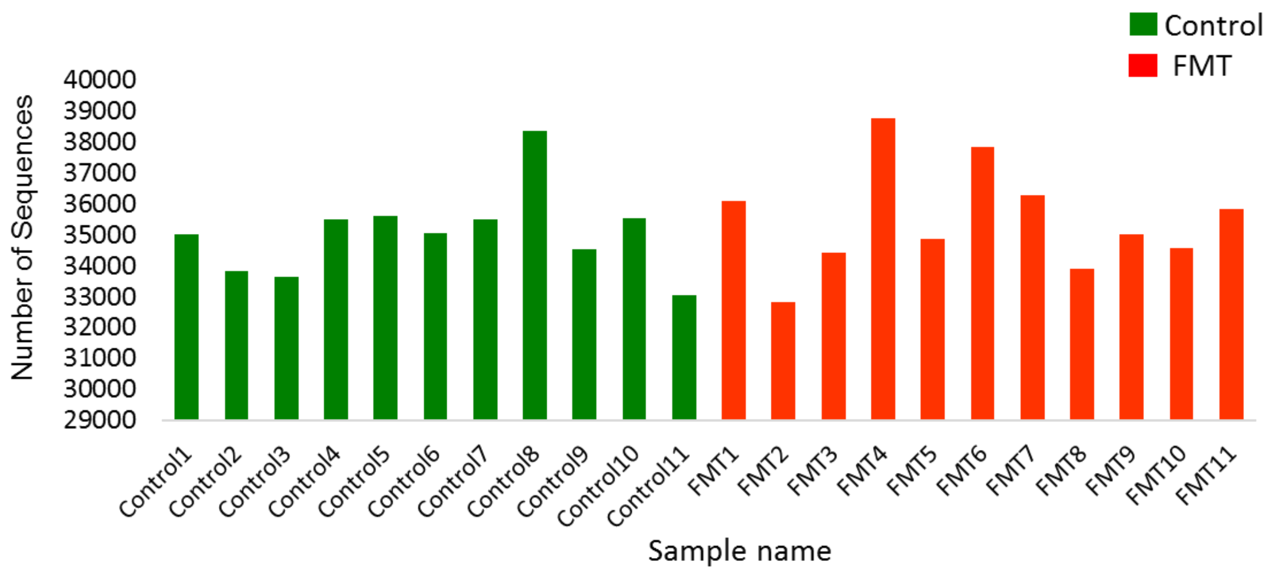

Figure S2. The total effective sequence amount that passed the quality screening and their indexes are perfectly matched.

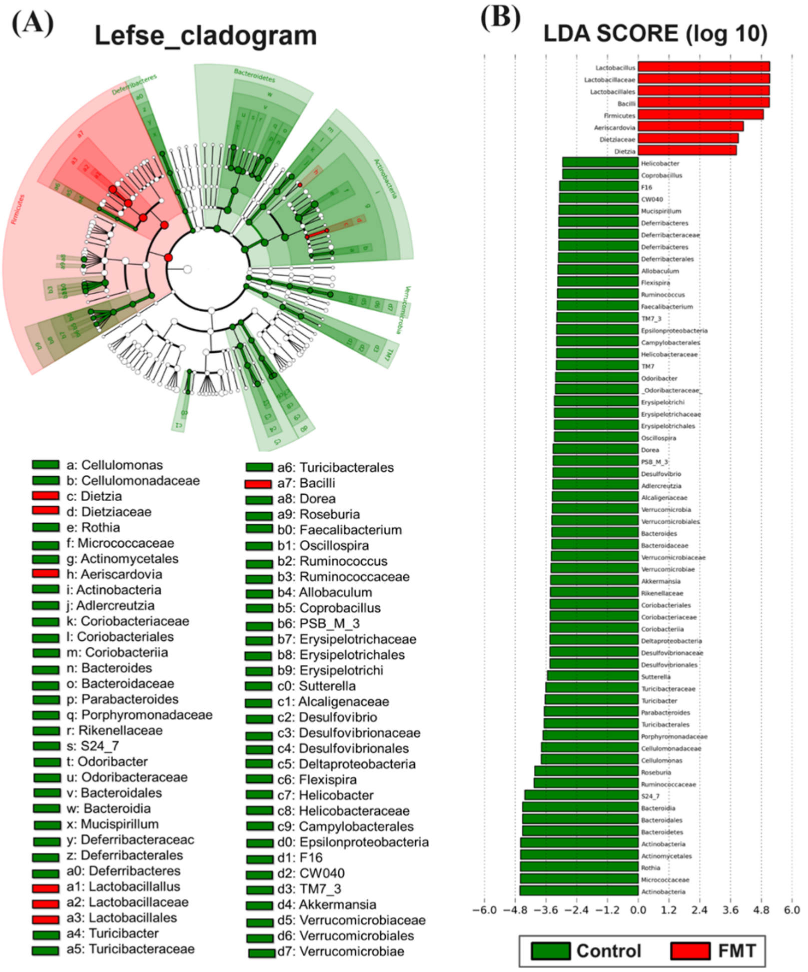

Figure S3. Taxonomic cladogram from LEfSe and LED scoreof the control and FMT groups. (A) Taxonomic cladogram from LEfSe showing differences in cecal taxa of cecum in control (green) microbiota and taxa enriched in FMT (red) of microbiota (*n* =11/group), the background color indicates phylum; names of phyla are indicated. Dot size is proportional to the abundance of the taxon. Letters correspond to the following taxa: a: *Cellulomonas*; b: *Cellulomonadaceae*; c: *Dietzia*; d: *Dietziaceae*; e: *Rothia*; f: *Micrococcaceae*; g: *Actinomycetales*; h: *Aeriscardovia*; i: *Actinobacteria*; j: *Adlercreutzia*; k: *Coriobacteriaceae*; l: *Coriobacteriales*; m: *Coriobacteriia*; n: *Bacteroides*; o: *Bacteroidaceae*; p: *Parabacteroides*; q: *Porphyromonadaceae*; r: *Rikenellaceae*; s: *S24_7*; t: *Odoribacter*; u: *Odoribacteraceae*; v: *Bacteroidales*; w: *Bacteroidia*; x: *Mucispirillum*; y: *Deferribacteraceac*; z: *Deferribacterales*. (B) LDA score of groups-enriched taxa are shown significant at $p < 0.05$, taxa enriched between groups are different colored (control with green and FMTwith red).

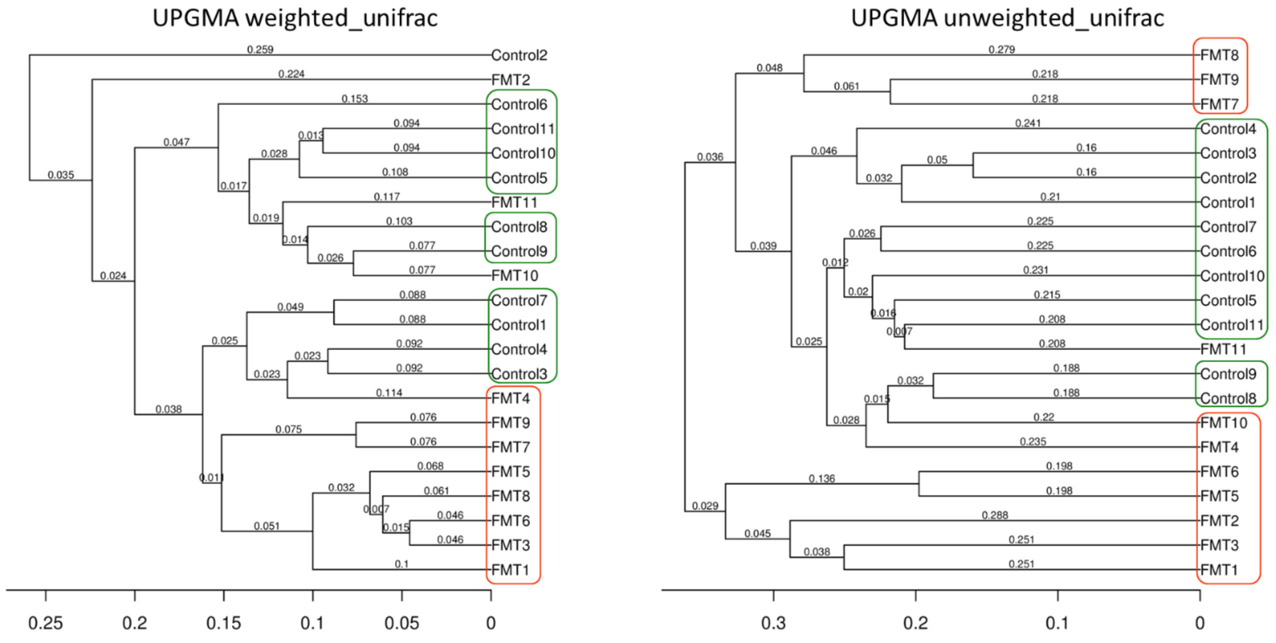

Figure S4. UPGMA clustering analysis was performed on unweighted and weighted unifrac distance matrices using QIIME software, and visualized using R software between groups of control and FMT of laying-chicks.

Table S1. Statistical number of OTUs at each classification level between control and FMT of laying-chicks.

Sample name	Phylum	Class	Order	Family	Genus	Species	Unclassified
Control1	1068	1068	1068	897	636	30	0
Control2	1036	1036	1036	836	496	35	0
Control3	1087	1087	1087	888	627	28	0
Control4	1274	1274	1274	1002	661	52	0
Control5	1020	1020	1020	850	687	59	0
Control6	985	985	985	860	626	63	0
Control7	1125	1125	1125	986	771	30	0
Control8	1076	1076	1076	923	669	59	0
Control9	963	963	963	819	622	42	0
Control10	858	858	857	785	627	56	0
Control11	848	848	848	733	558	49	0
FMT1	948	948	948	853	738	24	0
FMT2	478	478	478	453	397	10	0
FMT3	664	664	664	643	612	11	0
FMT4	1032	1032	1031	928	762	33	0
FMT5	453	453	453	451	447	5	0
FMT6	516	516	516	515	513	6	0
FMT7	702	702	702	653	582	34	0
FMT8	701	701	701	665	626	17	0
FMT9	666	666	665	619	538	38	0
FMT10	859	859	859	755	594	38	0
FMT11	953	953	953	806	582	39	0

Table S2. Statistical microbial community annotated at each classification level between control and FMT of laying-chicks.

Sample name	Phylum	Class	Order	Family	Genus	Species	Unclassified
Control1	9	17	22	40	41	11	9
Control2	9	16	22	36	41	11	9
Control3	9	16	21	33	36	9	9
Control4	9	17	24	36	46	14	9
Control5	10	21	27	45	53	19	10
Control6	9	17	24	42	49	15	9
Control7	9	17	21	42	50	18	9
Control8	12	21	30	53	75	31	12
Control9	12	21	31	54	68	28	12
Control10	12	23	35	60	77	30	12
Control11	10	19	27	50	59	20	10
FMT1	8	15	21	36	40	13	8
FMT2	5	11	12	16	14	6	5
FMT3	6	11	14	24	20	7	6
FMT4	9	17	25	38	51	14	9
FMT5	5	9	10	11	10	4	5
FMT6	5	9	11	13	11	3	5
FMT7	10	19	31	54	65	26	10
FMT8	6	12	17	31	34	11	6
FMT9	9	17	26	43	63	27	9
FMT10	9	18	27	51	63	27	9
FMT11	11	20	32	59	74	29	11
